# Short-Term Effects of Heat on Mortality and Effect Modification by Air Pollution in 25 Italian Cities

**DOI:** 10.3390/ijerph15081771

**Published:** 2018-08-17

**Authors:** Matteo Scortichini, Manuela De Sario, Francesca K. de’Donato, Marina Davoli, Paola Michelozzi, Massimo Stafoggia

**Affiliations:** 1 Department of Epidemiology, Lazio Regional Health Service, ASL Roma 1, 00147 Rome, Italy; m.scortichini@deplazio.it (M.S.); m.desario@deplazio.it (M.D.S.); f.dedonato@deplazio.it (F.K.d.D.); m.davoli@deplazio.it (M.D.); p.michelozzi@deplazio.it (P.M.); 2 Institute of Environmental medicine, Karolinska Institutet, Box 210, SE-171 77 Stockholm, Sweden

**Keywords:** air pollution, heat, air temperature, mortality, effect modification

## Abstract

Evidence on the health effects of extreme temperatures and air pollution is copious. However few studies focused on their interaction. The aim of this study is to evaluate daily PM10 and ozone as potential effect modifiers of the relationship between temperature and natural mortality in 25 Italian cities. Time-series analysis was run for each city. To evaluate interaction, a tensor product between mean air temperature (lag 0–3) and either PM10 or ozone (both lag 0–5) was defined and temperature estimates were extrapolated at low, medium, and high levels of pollutants. Heat effects were estimated as percent change in mortality for increases in temperature between 75th and 99th percentiles. Results were pooled by geographical area. Differential temperature-mortality risks by air pollutants were found. For PM10, estimates ranged from 3.9% (low PM10) to 14.1% (high PM10) in the North, from 3.6% to 24.4% in the Center, and from 7.5% to 21.6% in the South. Temperature-related mortality was similarly modified by ozone in northern and central Italy, while no effect modification was observed in the South. This study underlines the synergistic effects of heat and air pollution on mortality. Considering the predicted increase in heat waves and stagnation events in the Mediterranean countries such as Italy, it is time to enclose air pollution within public health heat prevention plans.

## 1. Introduction

Numerous epidemiological studies have reported the acute effects of air pollutants on mortality for non-accidental and cardiopulmonary causes [1,2]. Similarly, there is literature showing a significant and immediate (0–3 days) association between heat and daily mortality, with non-linear effects of high summer temperatures or linear above a threshold [3,4]. Results are consistent across different geographical areas, although some heterogeneity exists depending on local population characteristics and climatic conditions [2,4,5]. Furthermore, the same vulnerable population subgroups, such as the elderly and patients with cardio-respiratory disease and diabetes, have been identified in relation to short-term effects of heat or air pollution [6,7,8,9,10].

It is common practice in epidemiological time series studies to adjust for daily temperature when investigating the relationship between air pollution [11] and, to a lesser extent, control for daily air pollution levels in the analysis of extreme temperatures effects [12].

In contrast, the interactive or synergistic effects of the two environmental exposures on mortality has been less investigated. In the last decade, several studies focused on this aspect, mostly by providing effects of air pollutants stratified by season [13,14] and showing the highest effects in summer, especially of PM and O_3_ [15,16,17]. They all agree that the two exposures, air temperature and air pollution (either PM or O_3_), display a synergistic effect on mortality, with joint exposures increasing daily mortality risk more than the sum of the two individual effects. However, the shape and mechanisms of such interactions remain unclear [18].

While temperature has been shown to modify the air pollution-mortality relationship [16,17], much less is known on the potential effect modification induced by daily air pollutants on the association between air temperature and mortality in the warm season [19]. To our knowledge, this has never been investigated in Italy and only marginally elsewhere [19,20,21,22].

Recently, the fifth Assessment Report of the Intergovernmental Panel on Climate Change (IPCC) has identified the Mediterranean area as one of the most vulnerable hot spots to climate change for the 21st century [23]. Italy is particularly at risk due to the dominant high pressure during summer, which is associated with heat waves and the build-up of pollutants that entail the exceedance air quality standards especially in larger urban areas [24]. The objective of the study is to investigate the joint relationship between heat and air pollutants concentrations (PM and ozone) on daily mortality in 25 major Italian cities during summer. To address this aim, only deaths occurring in the warm season (defined as April to September) were selected. We estimated city-specific joint exposure-response curves of the two environmental risk factors (temperature and PM or ozone in turn) on daily non-accidental mortality, and we pooled the results into a meta-analysis where the association between summer temperature and mortality by low, medium, and high levels of the two air pollutants was reported. This aspect is of particular relevance in terms of public health prevention measures in urban areas during summer as heat waves and high levels of pollution are likely to occur concurrently, and adequate measures should be implemented to tackle both issues with particular focus on subgroups at risk.

## 2. Materials and Methods

### 2.1. Dataset

Data was collected for 25 Italian cities (Ancona, Bari, Bologna, Brindisi, Cagliari, Ferrara, Firenze, Genova, Mestre-Venezia, Milano, Modena, Napoli, Padova, Palermo, Parma, Piacenza, Pisa, Reggio Emilia, Rimini, Roma, Rovigo, Taranto, Torino, Treviso, and Trieste) included in the national project EPIAIR2 [1]. Figure S1 shows the geographical distribution of the 25 cities included in the study: the different colors represent the three areas—North, Center, and South. All cities are larger than 80,000 inhabitants (except Rovigo, with circa 50,000 residents). For each city daily mortality, meteorological and air pollution data for the period 2006–2010 was provided depending on availability.

Briefly, the outcome variable considered in this study was the city-specific daily mortality count for non-accidental causes (International Classification of Diseases 9th or 10th version [ICD-9] 001-799 or [ICD-10] A00-R99) among subjects of age 35+ years residing and dying in each of the 25 cities.

Temperature data was retrieved from the airport station closest to each city. Daily mean temperature (°C) was calculated as the average of the 6-h temperature readings in the 24 h period.

The air pollutants considered in the analysis were PM10 and O_3_. The regional air quality monitoring networks provided, for each city, the daily time series of pollutants, retrieved from urban monitors. Only monitors meeting the requirements of EPIAIR2 protocol were included in the analysis [25]. The same protocol was applied to impute missing values for each monitor and to derive daily mean concentrations for PM10, and daily maximum 8-h running means for O_3_.

### 2.2. Statistical Methods

We ran an over-dispersed Poisson generalized additive regression model in each city, as the following:

log[E(Y_i)] = α + tensor(〖Tmean〗_(0–3), 〖Pollutant〗_(0–5)) + s(time) + s(dos)+ dow + hol + pop_hol

where Y_i is the number of non-accidental deaths in the day i; Tmean0–3 is a moving average of the daily mean temperature of the current day and previous three days; Pollutant0–5 is a moving average of the daily concentration of air pollutant considered in the model (either PM10 or O_3_) of the current day and previous five days; Time is the progressive count of days in the study period, modelled with a spline with a number of degrees of freedom (d.o.f.) equal to the city specific number of years available; dos is the day of season (values from 1 to 183) fitted with a spline with six d.o.f. (one for each month) to control for sub-seasonal trends; dow and hol are categorical variables for day of the week and holidays, respectively; and pop_hol is a categorical variable accounting for population decrease during the summer vacation periods (it assumes value “2” in the two-week period around mid-August, value “1” between mid-July and 31st of August (with the exception of the aforementioned two-week period), and value “0” otherwise). The lag windows for air temperature and air pollutants were defined a priori, based either on literature [26] or on previous investigations on the same dataset [1].

The interaction between temperature and air pollution was analyzed by running a “response surface” model: this methodology allows us to represent the combined relationship between two risk factors and the outcome of interest with a non-parametric approach by defining a tensor smoother, i.e., a tridimensional curve modeling the increases in mortality according to a combined variation of the values of temperature and air pollutant. The tensor was estimated by fitting a cubic spline with four d.o.f. for temperature and a cubic spline with three d.o.f. for pollutants.

A random-effects meta-analysis was run to obtain pooled estimates at country level and by geographic areas, defined as North (Treviso, Trieste, Milano, Venezia, Padova, Rovigo, Torino, Piacenza, Ferrara, Parma, Reggio Emilia, Modena, Bologna, Genova, Rimini), Center (Firenze, Pisa, Ancona, Roma), and South (Bari, Napoli, Brindisi, Taranto, Cagliari, Palermo). The effect of heat was estimated as the % change in mortality, with 95% confidence intervals (95% CI), for an increase in mean temperature from the 75th to the 99th percentile of the city-specific distribution of mean temperature.

In order to evaluate if the effect of temperature was modified by air pollution, three levels of air pollution considered as “low,” “medium,” and “high” were defined for each city, equals to the 5th, 50th and 95th percentile of the city-specific air pollutants distributions. Then, from the tridimensional surface estimated in each city, three dose-response curves were extrapolated along the aforementioned values of pollutants, expressing the relationship between temperature and mortality corresponding to low, medium, and high pollution values. Finally, the % change in mortality was estimated from each curve, and 95% confidence intervals were calculated with a bootstrap procedure.

**Figure 1:**
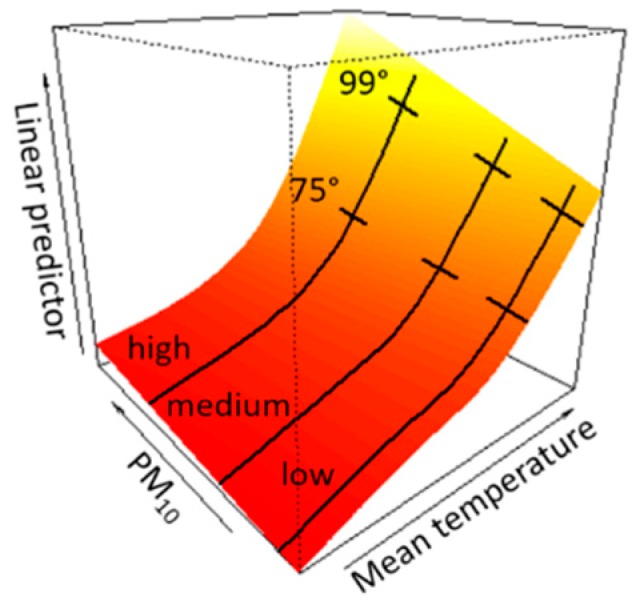
Example of the tensor smoother depicting the joint non-linear association between mean temperature and PM10 on non-accidental mortality. The graph shows the percentiles chosen to show effect estimates of temperature (75th and 99th percentiles of temperature distribution) and the sections corresponding to low, medium and high pollution values (5th, 50th, and 95th percentiles of PM10 distribution).

A graphical example of the tensor smoother, with temperature effects stratified by pollutants values, is displayed in Figure 1. The graph shows the percentiles chosen to express effect estimates of temperature (75th and 99th percentiles of temperature distribution) and the sections corresponding to low, medium and high pollution values (5th, 50th, and 95th percentiles of PM10 distribution).

To test for the presence of effect modification by air pollution, the Cochran Q test and I^2^ statistic were estimated on the effects pooled by geographic area.

All analyses were conducted using the packages mgcv, metafor.in R statistical software [27].

## 3. Results

Study cities average population varies from 52,118 residents in Rovigo to 2,743,796 in Roma (Table S1). Considering the three geographic areas, total population is around 5 million in the North, 3.3 million in the Center and 2.4 million in the South. There is a clear South-North gradient in the proportion of elderly residents (65+), ranging from 18.4% to 24.5%. The geographical gradient is steeper among the very old (85+).

Table 1 shows, for each city, the study period, total and daily deaths in the 35+ years population, mean and standard deviation of air temperature distributions, and the percentiles used to estimate the effect of heat (75th and 99th percentiles) and the concentrations of pollutants used to define low, medium, and high levels of PM10 and O_3_ by city. According to this study, 187,743 subjects aged 35+ years died from non-accidental causes in the study period in the 25 cities, with a high heterogeneity in the daily number of deaths (from 1.3 in Rovigo to 54.0 in Roma). A high variability was also observed for mean temperature (19.7 °C in Torino, 23.1 °C in Palermo), with an increasing North to South gradient also observed in the 75th and 99th percentiles of the city-specific temperature distributions. For air pollutants, no major geographical patterns were detected, with highest PM10 values in the Po Valley area (Milano, Venezia, Padova, Torino) but also in central and southern major cities (Firenze, Roma, Napoli, Bari, and Palermo).

**Figure 2:**
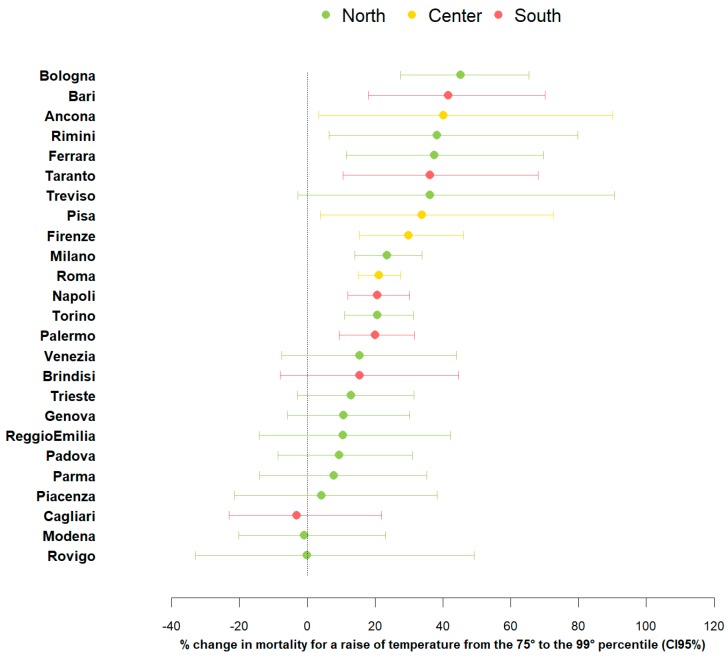
City-specific estimates of the heat effects. Estimates are given as % change in non-accidental mortality, and 95% confidence intervals (95% CI) for mean air temperature increases between the 75th and 99th percentile of city-specific temperature distributions.

City-specific heat effects are shown in Figure 2. A significant effect of heat on mortality is observed in most cities for temperature increases between the 75th and 99th percentile of the mean temperature distributions. Furthermore, no clear geographical trend is observed, with the highest effects of heat in Bologna, Bari, and Ancona, respectively, from the North, South, and central regions. The overall meta-analytical effect is significant (results not shown), with similar magnitudes in southern (% change +15.2; 95% CI: 8.4–22.3 for temperature increases from 75th to 99th percentile) and central cities (% change +15.1; 95% CI: 8.8–21.8) and slightly lower effects observed in northern cities (% change +10.1; 95% CI: 7.2–13.2).

**Figure 3:**
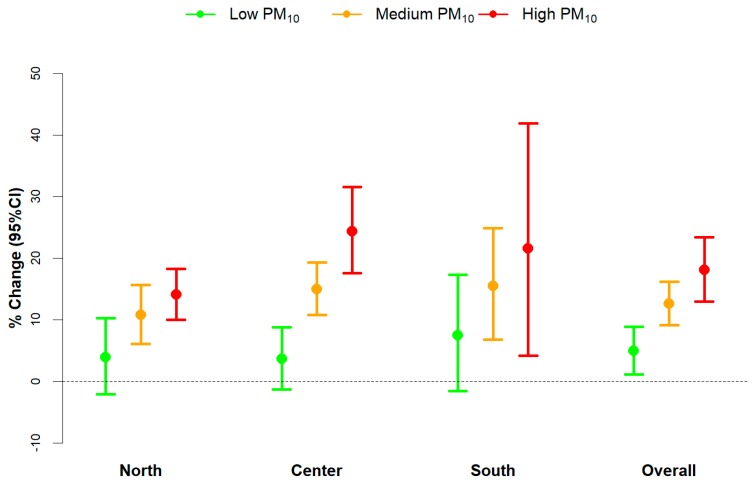
Area-specific (North, Center, South) and overall meta-analytical estimates of the heat effects, stratified by levels of PM10. Estimates are given as % change in non-accidental mortality, and 95% confidence intervals (95% CI) for mean air temperature increases between the 75th to 99th percentile of city-specific temperature distributions, by levels of PM10 (defined as “low”, “medium”, and “high” based on 5th, 50th, and 95th percentiles of city-specific PM10 distributions).

**Figure 4:**
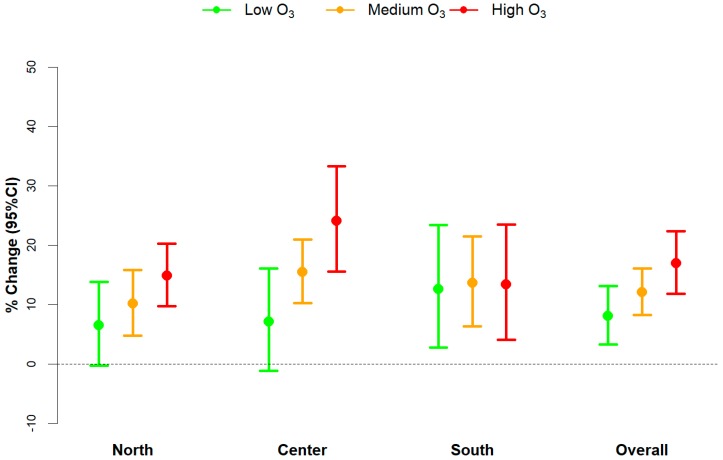
Area-specific (North, Center, South) and overall meta-analytical estimates of the heat effects, stratified by levels of O_3_. Estimates are given as % change in non-accidental mortality, and 95% confidence intervals (95% CI) for mean air temperature increases between the 75th to 99th percentile of city-specific temperature distributions, by levels of O_3_ (defined as “low”, “medium”, and “high” based on 5th, 50th, and 95th percentiles of city-specific O_3_ distributions).

Figure 3 and Figure 4 show area-specific (North, Center, South) and overall meta-analytical estimates of the effect of heat, stratified by levels of PM10 and O_3_ respectively (city-specific estimates are reported in supplementary material, Tables S2 and S3). In the North, a raise in mean temperature (from 75th to 99th percentile) caused an increase in mortality of +3.9% (95% CI: −2.1–10.2; I^2^ = 43%) on days with low levels of PM10, while on days characterized by medium or high concentrations, the increases were equal to +10.8% (95% CI: 6.1–15.6; I^2^ = 58%) and +14.1% (95% CI: 10.0–18.3 I^2^ = 0%), respectively. In the Center, the trend was even steeper: the increases in temperature-related mortality rose from +3.6% (95% CI: −1.3–8.8 I^2^ = 0%) to +14.9% (95% CI: 10.7–19.3 I^2^ = 10%) and +24.4% (95% CI: 17.6–31.6 I^2^ = 15%) for days with low, medium, and high PM10 concentrations, respectively. A similar trend was found in southern cities, with estimates ranging between +7.5% (95% CI: −1.6–17.3 I^2^ = 25%) to +15.5% (95% CI: 6.8–24.9 I^2^ = 54%) and +21.6% (95% CI: 4.2–41.9 I^2^ = 82%). The overall estimates on all the 25 cities confirms the increasing trend observed in the area-specific results.

Corresponding results for the effect modification by O_3_ are displayed in Figure 4. We found increasing effects of temperature for increasing levels of ozone in the north, with a +6.5% change in mortality (95% CI: −0.3–13.8 I^2^ = 64%) for low, +10.1% (95% CI: 4.8–15.8 I^2^ = 65%) for medium, and +14.8% (95% CI: 9.7–20.2 I^2^ = 39%) for high levels of O_3_. The results for central cities showed a similar pattern, with temperature estimates for increasing O_3_ concentrations equal to +7.1% (95% CI: −1.1–16.1 I^2^ = 0%), +15.5% (95% CI: 10.2–21.0 I^2^ = 0%), and +24.1% (95% CI: 15.6–33.3 I^2^ = 32%), respectively. In contrast, no effect modification was observed in southern cities, the increase in temperature-related mortality being constant among the three levels of air pollution (+12.6%; 95% CI: 2.8–23.4 I^2^ = 55%, +13.6%; 95% CI: 6.3–21.5 I^2^ = 67%, +13.4%; 95% CI: 4.1–23.5 I^2^ = 60% from low to high O_3_ values). The overall pooled analysis showed a positive interaction but was more contained compared to the one estimated for PM10.

## 4. Discussion

The present study investigated the joint relationship between summer temperatures and air pollutants on daily mortality in 25 Italian cities using innovative approaches for estimating non-linear associations and effect modification. We confirmed a high and significant effect of high summer temperatures on daily mortality. City-specific tensor smoothers showed increased risks per increasing levels of both exposures, suggesting a synergistic effect on mortality, i.e., a joint effect greater than the sum of individual effects. Finally, we detected an effect modification induced by both air pollutants (PM10 and O_3_) on the temperature-mortality association, with highest temperature-related mortality on days with high air pollution concentrations.

Our results can be compared with the existing evidence from the epidemiological literature, which analysed a synergy between extreme temperatures and high concentrations of air pollution in increasing daily mortality risks [18,19,20,22,28,29]. The evidence on effect modification induced by air pollution from previous studies is limited and with mixed results. Evidence for modification by particles and ozone was provided by a multicenter European study considering heat waves instead of temperature effects [20], while a large nine cities US study did not find any evidence of interaction between temperature and outdoor PM2.5 or ozone [30]. A study on Lisbon and Berlin found a different pattern of effect modification in the two cities by PM10 since the pollutant increased in summer in the first city and decreased in the second one [19], while both cities recorded a synergistic effect between high temperature and ozone.

The research question of the effect modification induced by air pollution in the temperature-mortality relationship is worth considering the heat related effects are large compared to air pollution, therefore the study of the potential modification has a greater public health relevance especially since heat response plans are in place in many cities but virtually none includes information on air pollution [31]. Air pollutants may have synergistic effects with high temperature in different ways, by increasing exposure levels in the atmosphere, by enhancing the intake of pollutants within the human body and through interconnected pathogenetic pathways. The first level of interaction is depicted by the air pollution peaks occurring during stagnation events common in summer heat waves [32,33] and by the photochemical reactions producing ozone in the atmosphere under conditions often related to heat waves (high solar irradiance and high temperature). Moreover, it has been suggested that during the warmer months, when people spend more time outdoors or keep windows open, exposure to atmospheric pollutants and heat are greater with potential detrimental effects on health, especially among the most vulnerable subgroups [15]. The second type of interactions is due to the activation of thermoregulatory mechanisms such as the increase in ventilation rate that consequently increase the intake of air pollutants into the airways [34]. The third level of interaction is within the body especially in vulnerable subjects but mechanisms are little known. A possible explanation is that thermoregulatory responses to heat stress conditions may reduce the ability of the body to detoxify chemicals [34], but specific pathways are possibly triggered by ozone and by particles interactive effects. Ozone has a potent oxidant toxicity for the lung and impairs host defenses against respiratory infections, further aggravating the heat-related effects on the body [35]. Particulate matter seems to activate the same cellular pathways than high temperature, e.g., growing levels of markers of systemic inflammation such as C-reactive protein, by potentiating temperature effects [36,37].

Our study has a national representativeness and allows to evaluate geographical heterogeneity of temperature-air pollution interactions. The largest interactions were in the central and northern cities. Such geographical differences do not rely on air pollution effects heterogeneity [1], but they could be explained by local climatic conditions affecting population capacity to cope with different summer temperature ranges and by differences in population susceptibility (e.g., % 65+ years old population has an inverse North-South gradient in Italy ranging from 18% in the South to 25% in the North).

This is one of the few studies which hypothesized a possible effect modification induced by air pollutants in the temperature-mortality relationship and found evidence of a higher temperature-related mortality risk on very polluted days. This result has important consequences, as the frequency of extreme meteorological conditions will increase over time, and there is evidence of stable air pollution effects documented even at low concentrations. This has been predicted in the Mediterranean hot spot [32,33]. Our study confirms a North-South gradient in heat effects; southern areas are highly exposed to heat stressful conditions in summer and scenarios for the Mediterranean basin show a disproportionate increase in heat wave duration and intensity compared to the other European regions [23]. Furthermore an increase in stagnation episodes in the same area is expected, favoring photochemical reactions and increasing persistence of pollutants [38]. Both environmental exposures especially affect the most vulnerable subgroups, such as the elderly and those with pre-existing cardio-respiratory conditions [6,7]. Furthermore, it is known that the elderly population has been increasing over the last decades [39], and it represents a pool of susceptible individuals upon which prevention measures should be targeted to, in order to minimize the joint impact of extreme temperatures and air pollution.

This paper highlights the relevance of the acute effects of heat and air pollution, that should be accounted for when calculating the whole health burden, in addition to the long-term effects which are very serious for air pollution, classified as carcinogenic to humans (Group 1) by the International Agency for Research on Cancer [38].

Strengths of the study include the fact it assesses whether daily air pollution might modify the short-term association between summer temperature and mortality in 25 cities, representative of diverse geographical and climatic conditions. Secondly, the use of tridimensional non-parametric curves allowed us to relax linearity assumptions often underlying time-series studies, while providing more flexible and robust estimates. Among the weaknesses of the study, it is important to highlight the ecological study design, which assumes the exposure is the same over the entire spatial domain (city) which is a limit in all time-series studies. The study only accounts for urban areas as both temperature and air quality monitors are lacking in sub-urban and rural settings in Italy. Another limitation is that only mortality for all natural causes was used; this choice was made since we modeled the interaction with a bivariate tensor smoother, requiring a considerable number of daily outcome counts in order to converge.

## 5. Conclusions

The present study underlines the importance of jointly investigating the short-term health effects of air pollution and air temperature in order to fully capture the health effects during summer. In Italy, high and significant effects of heat on daily mortality in summer are shown. Furthermore, although there are some geographical differences, the risk in heat-related mortality is greatest when high levels of PM10 and ozone occur. Since future vulnerability to both heat waves and air pollutants is expected to become a major concern in the Mediterranean countries such as Italy [23,24,32,33], in light of the synergies between the two exposures, heat adaptation and response plans should incorporate such interactions into warning systems and prevention measures especially among vulnerable subgroups.

## Figures and Tables

**Table 1:** Description of city-specific data: study period, mortality data in 35+ years population, air temperature distribution and percentiles of air pollutants used to define days with low, medium, and high PM10 and O_3_ values.

Area	City	Study Period	Mortality for Natural Causes		Mean Temperature				PM_10_			O_3_		
			*n*.	Daily Mean	Mean	SD	75° pctile	99° pctile	5° pctile	50° pctile	95° pctile	5° pctile	50° pctile	95° pctile
North	Treviso	2006–2010	3202	2.2	13.8	7.9	23.8	29.1	5.0	24.0	45.7	55.6	103.8	158.9
	Trieste	2006–2010	12,818	7.0	15.7	7.0	24.6	28.9	7.5	20.0	39.6	64.8	97.3	136.8
	Milano	2006–2010	51,710	28.3	13.9	8.3	24.7	29.0	12.0	28.6	51.5	45.7	92.1	150.4
	Venezia	2006–2009	6827	4.7	14.3	8.0	24.5	29.0	11.1	30.0	59.0	56.4	92.7	138.2
	Padova	2006–2009	8640	5.9	13.7	8.2	24.1	28.6	10.0	31.5	58.1	57.4	106.8	162.6
	Rovigo	2006–2010	2050	1.4	13.7	8.0	23.8	27.6	11.0	24.0	46.0	70.3	112.6	161.0
	Torino	2006–2010	37,104	20.4	12.9	8.3	23.3	27.7	10.5	27.5	53.9	60.3	105.4	161.1
	Piacenza	2006–2010	5019	2.7	14.4	8.5	25.3	29.2	10.5	25.0	46.4	51.3	102.7	158.3
	Ferrara	2006–2010	7875	4.3	14.6	8.3	25.2	29.6	11.0	24.0	43.0	71.1	109.3	153.0
	Parma	2006–2010	8545	4.7	15.2	8.4	26.0	30.4	10.7	24.0	45.6	56.9	105.0	150.3
	Reggio Emilia	2006–2010	6519	3.6	14.8	8.4	25.7	30.2	11.7	25.0	46.1	61.1	107.6	162.3
	Modena	2006–2010	8599	4.7	14.6	8.4	25.5	30.0	12.7	26.3	48.8	56.4	106.1	158.7
	Bologna	2006–2010	19,223	10.5	14.7	8.6	25.9	30.6	14.0	26.0	47.0	44.9	91.8	148.0
	Genova	2006–2010	14,623	20.0	15.8	6.6	24.0	28.2	14.2	25.2	41.6	64.7	99.5	134.4
	Rimini	2006–2010	5724	3.1	15.1	7.6	24.6	29.3	12.5	25.2	43.2	59.1	92.9	132.1
Center	Firenze	2006–2010	15,082	10.3	15.5	7.2	24.9	28.8	17.4	30.1	49.2	66.1	104.2	143.3
	Pisa	2006–2010	3513	2.4	15.0	6.7	23.5	27.3	15.6	28.2	44.0	65.9	95.2	126.5
	Ancona	2006–2010	4447	2.4	14.8	7.1	23.9	28.8	15.2	28.9	51.3	43.2	85.7	115.5
	Roma	2006–2010	104,795	57.4	15.9	7.0	25.4	29.4	17.3	30.3	50.0	62.7	95.1	134.2
South	Bari	2006–2010	4812	6.6	16.1	6.8	25.0	30.8	14.8	28.4	52.0	64.4	98.4	126.1
	Napoli	2006–2009	33,443	22.9	16.9	6.5	25.7	29.0	12.4	30.6	59.2	45.6	100.6	141.0
	Brindisi	2006–2010	2404	1.6	17.6	6.5	26.5	30.1	7.1	22.5	44.5	78.1	105.3	133.1
	Taranto	2006–2010	5881	4.0	17.5	6.7	26.8	30.8	13.3	26.4	52.9	80.1	110.7	145.5
	Cagliari	2006–2010	6355	3.5	17.4	6.0	25.5	29.0	13.1	24.3	44.2	47.1	69.7	92.4
	Palermo	2006–2009	22,116	15.1	18.8	6.0	26.7	31.0	19.8	32.9	63.9	56.1	79.8	102.1

SD: Standard Deviation

## Supplementary Materials

The following are available online at http://www.mdpi.com/1660-4601/15/8/1771/s1. Table S1: City-specific all ages resident population by age groups (average 2001–2010). Table S2: City-specific (North, Center, South) estimates of the heat effect, stratified by levels of PM10. Estimates are expressed as % change in non-accidental mortality, and 95% confidence intervals (95% CI) per increases of mean air temperatures from 75th to 99th percentile of city-specific temperature distributions, by levels of PM10 (defined as “low,” “medium,” and “high” based on 5th, 50th, and 95th percentiles of city-specific PM10 distributions). Table S3: City-specific (North, Center, South) estimates of the heat effect, stratified by levels of ozone (O_3_). Estimates are expressed as % change in non-accidental mortality, and 95% confidence intervals (95% CI) per increases of mean air temperatures from 75th to 99th percentile of city-specific temperature distributions, by levels of O_3_ (defined as “low,” “medium,” and “high” based on 5th, 50th, and 95th percentiles of city-specific O_3_ distributions).
