# Supplementary figures and images for: Short-Term Effects of Heat on Mortality and Effect Modification by Air Pollution in 25 Italian Cities
Source: Int J Environ Res Public Health. 2018 Aug 17;15(8):1771. doi: 10.3390/ijerph15081771 (PMC6122066; doi:10.3390/ijerph15081771)

**Figure S1. Geographical distribution of the 25 cities in the study, divided by macro-area.**


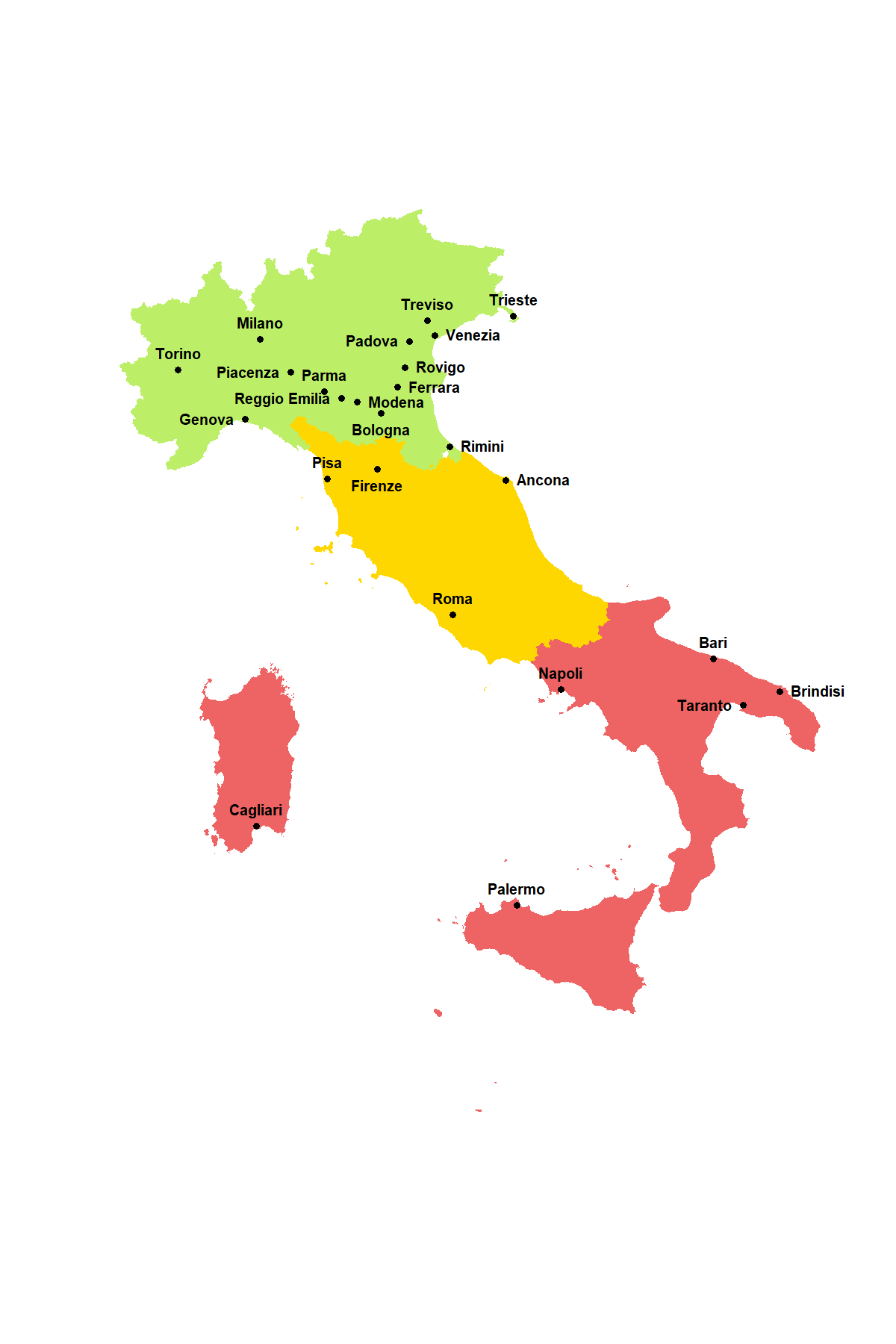

Supplement: Supplementary file 1 [file ijerph-15-01771-s001.zip › Table S1 S2 S3, Figure S1/Figure S1.docx]
